# Supplementary material for: Stoichiometry and Thickness of Epitaxial SrTiO3 on Silicon (001): An Investigation of Physical, Optical, and Electrical Properties
Source: Cryst Growth Des. 2025 Jul 22;25(15):5752–60. doi: 10.1021/acs.cgd.5c00103 (PMC12333012; doi:10.1021/acs.cgd.5c00103)
Supplement: Supplementary file 1 [file cg5c00103_si_001.pdf]

Supporting information for

Stoichiometry and Thickness of Epitaxial $SrTiO_3$ on Silicon (001): an Investigation of Physical, Optical and Electrical Properties

Andries Boelen[1,2], Marina Baryshnikova[1], Anja Ulrich[1,3], Kamal Brahim[1,4], Joris Van de Vondel[5], Christian Haffner[1] and Clement Merckling[1,2]

1) Imec, B-3001 Leuven, Belgium
2) Department of Materials Engineering (MTM), KU Leuven, B-3001 Leuven, Belgium
3) Department of Information Technology (INTEC), Photonics Research Group, Ghent University, B-9052 Ghent, Belgium
4) Department of Electrical Engineering (ESAT), KU Leuven, B-3001 Leuven, Belgium
5) Department of Physics and Astronomy, KU Leuven, B-3001 Leuven, Belgium

Appendix A: Supporting figures

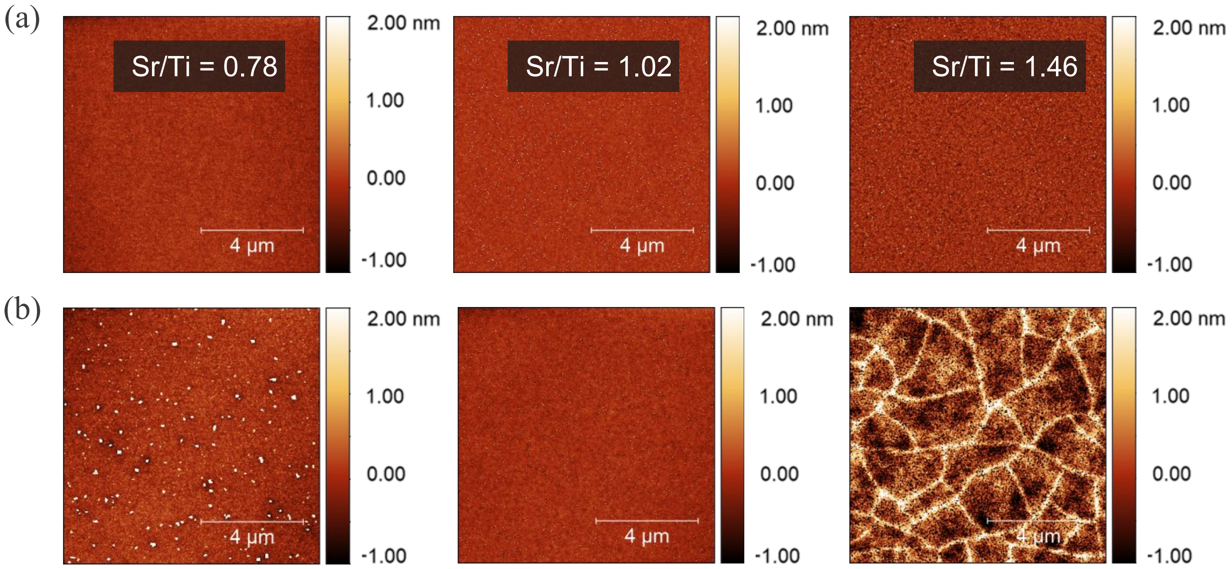

Supplementary Figure 1: 10 x 10 µm AFM images for the three STO samples with varying cationic stoichiometry. (a) As-grown STO samples all show a smooth surface (R_q < 0.4 nm). (b) STO after PGA 850°C 60 min. Large particle formation causes the surface roughness of Ti-rich STO to increase, whereas deep cracks and pinholes severely degrade the surface of Sr-rich STO.

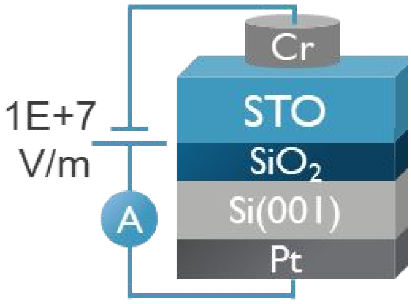

Supplementary Figure 2: Schematic of the Cr/STO/SiO_2/Si/Pt stack used for I-V measurements applying 1E+7 V/m. The circular top contact has a radius of 200 µm.

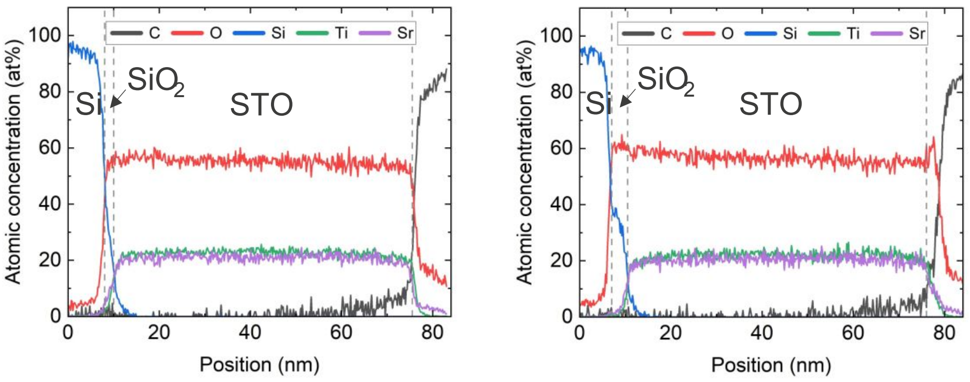

Supplementary Figure 3: EDS corresponding to TEM in Figure 7 before (left) and after PGA (right). Both Sr and Ti atomic concentrations are constant throughout the film and the interfacial SiO_2 layer increases after PGA.

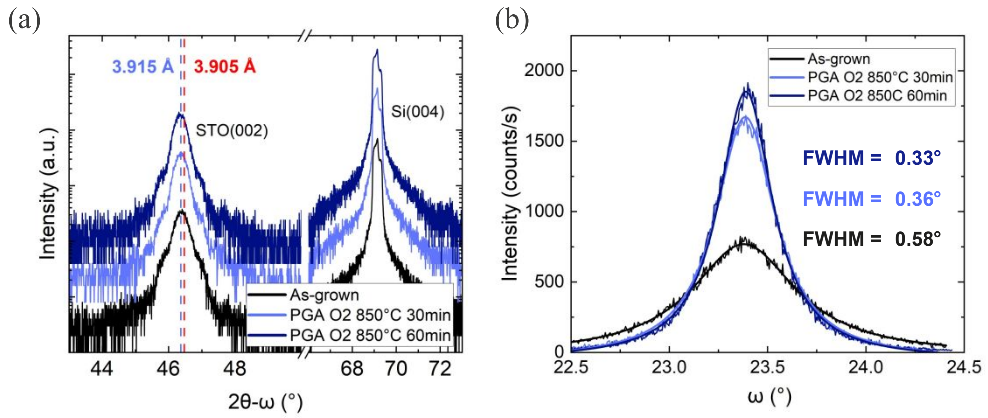

Supplementary Figure 4: As an example of the XRD results, the 26 nm stoichiometric STO film is shown before and after PGA. (a) 2θ-ω scan showing the STO (002) and Si (004) diffraction peaks. The out-of-plane lattice parameter of the film after PGA for 30 min (light blue) and bulk (red) are indicated by dashed vertical lines. (b) ω scan shows the decreasing FWHM after PGA, indicating improved crystallinity.

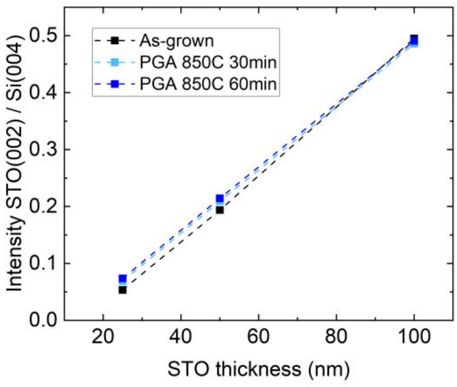

Supplementary Figure 5: XRD intensity of the STO (002) diffraction peak as a function of STO thickness before and after PGA showing a linear increase as expected.

Appendix B: Ellipsometry fitting

The model used for fitting ellipsometry data consists of five Tauc-Lorentz oscillators and includes both an intermediate silicon-oxide SiO_x layer and a SiO_2 layer between the STO and Si to model the silicon oxidation. AFM surface roughness from the 10 x 10 µm scan was used to define the range for the fitted roughness, with a margin of ~ 0.1 nm from the AFM value.

The mean squared error (MSE) of the fitting is calculated by the CompleteEASE software using the following equation:

$$MSE = \sqrt{\frac{1}{3n-m}\sum_{i=1}^{n}\left[\left(\frac{N_{E_i}-N_{G_i}}{0.001}\right)^2+\left(\frac{C_{E_i}-C_{G_i}}{0.001}\right)^2+\left(\frac{S_{E_i}-S_{G_i}}{0.001}\right)^2\right]}$$

where n is the number of wavelengths, m is the number of fitting parameters, and $N = \cos(2\Psi)$, $C = \sin(2\Psi i)\cos(\Delta)$, $S = \sin(2\Psi)\sin(\Delta)$. Subscript E and G denote measured and generated data, respectively.

For all our samples, MSE values are below 8, indicating a good fit. As-grown samples all have a fitted SiO_2 layer below 2.4 nm, whereas after PGA it is between 3.3 nm and 4.8 nm, in accordance with TEM. Only for the annealed Sr-rich STO the fit is worse due to the thick ~ 40 nm interfacial layer and high defectivity, resulting in an MSE of 25.4.
